# Micronutrients and pregnancy; effect of supplementation on pregnancy and pregnancy outcomes: *a systematic review*

**DOI:** 10.1186/1475-2891-12-20

**Published:** 2013-01-31

**Authors:** Taddese Alemu Zerfu, Henok Taddese Ayele

**Affiliations:** 1 College of Health Sciences and Referral Hospital, Dilla University, Dilla, Ethiopia; 2 Food Science and Nutrition Program, College of Natural Sciences, Addis Ababa University, , Ethiopia; 3 Department of Infectious Diseases Epidemiology, Devision of Julius Center for Health Sciences & Primary Care, University Medical Center, Heidelberglaan 100, Utrecht, 3584, CX, the Netherlands

## Abstract

**Introduction:**

Every year more than 20 million infants are born with low birth weight worldwide. About 3.6 million infants die during the neonatal period. More than one third of child deaths are thought to be attributable to maternal and child under nutrition.

**Objectives:**

To systematically review the effect of supplementing various combinations and types of micronutrients on the course and outcomes of pregnancy.

**Methods:**

Electronic search of Medline, Pub Med, Health Internetwork access to Research Initiative, and Google Scholar databases was conducted. Outcomes of interest were birth weight, low birth weight, small size for gestational age, prenatal mortality and neonatal mortality. After exclusion of irrelevant /incomplete ones, 17 out of 115 articles were considered for the final analysis.

**Findings:**

Majority of the articles reviewed favored the supplementation of micronutrients to pregnant mother. Some studies suggested calcium supplementation is associated with a significant protective benefit in the prevention of pre-eclampsia. The remaining articles reviewed, showed significant benefit of Multiple Micronutrients supplementation during pregnancy in reducing low birth weight, small for Gestational Age births as compared to the usual iron-folate supplements.

**Conclusions:**

Supplying micronutrients, mainly multiple micronutrients have beneficial effect in reducing the risk of low birth weight and other complications. Further studies at various combination and doses of micronutrient supplements are recommended.

## Introduction

Every year more than 20 million infants are born with low birth weight worldwide [1]. About 3.6 million infants die during the neonatal period [2]. Two thirds of these deaths occur in southern Asia and sub-Saharan Africa. More than one third of child deaths are thought to be attributable to maternal and child under nutrition [3]. Deficiencies in micronutrients such as folate, iron and zinc and vitamins A, B6, B12, C, E and riboflavin are highly prevalent and may occur concurrently among pregnant women [3]. Micronutrient deficiencies result from inadequate intake of meat, fruits and vegetables, and infections can also be a cause. Multiple micronutrient supplementations in pregnant women may be a promising strategy for reducing adverse pregnancy outcomes through improved maternal nutritional and immune status [4,5].

The World Health Organization (WHO) currently recommends iron and folic acid supplementation to reduce the risk of iron deficiency anemia among pregnant women. Since many developing countries already have systems in place for the delivery of iron and folic acid supplements, micronutrient supplements could be provided at little additional cost [6].

Several systematic reviews of trials examining the effects of maternal multiple micronutrient supplementation have been conducted [7-10] but they have had limitations. Although some researchers have raised concerns that micronutrient supplementation may increase perinatal mortality, none of the previous review articles has adequately addressed this issue [7-9,11]. None has examined the potential sources of heterogeneity in the effect of supplementation on perinatal mortality.

In addition, most of the studies and literature reviews dealing with maternal nutrition and birth outcomes have approached the issue by investigating single nutrients in isolation. On one level, this is necessary for an in-depth study of the complex issues involved. However, nutrient deficiencies are generally found in low-Socio-economic populations, where they are more likely to involve multiple rather than single deficiencies [4]; and studies that address and bring together the broader picture of multiple nutrient intakes or deficiencies are lacking [5].

The effects of maternal micronutrient supplementation on perinatal mortality and other pregnancy outcomes can differ depending on trial characteristics and study population. An updated systematic review is essential to provide the basis for future research and for a discussion of policy implications. Hence, this manuscript examines the effect of micronutrient supplements on the progress and outcomes of pregnancy by reviewing high quality literatures/articles.

## Methods

The published results from high-quality human observational and experimental studies which analyzed the effects of supplying Multiple Micro Nutrients (MMN) on the course of pregnancy and pregnancy outcomes were all included to this literature based analysis. Electronic search of Medline, Pub Med, Health Internetwork Access to Research Initiative (HINARI), and Google Scholar databases up to the end of 2011 was conducted. Search was done in keywords: (“Mothers” OR “Pregnancy” OR mother* OR maternal OR pregnancy) AND (“Micronutrients” OR “multiple micronutrient*” OR multivitamin OR micronutrient*) AND (supplement*) AND (Observational OR studies OR clinical trials). A function extracting related articles as well as reference lists from research, reviews and editorials was used during the search process. The full version of the English-language analyzed articles and abstracts of most found papers were available during the selection process except for pubmed search.

All literatures, including: observational studies, quasi-randomized trials and prospective randomized controlled trials (RCTs) evaluating multiple micronutrient supplementation in women during pregnancy, published in English language, were included. There were no limits on gestational age at the time of enrolment in the study and the duration of supplementation. Other than the assessment of Small for Gestational Age and neonatal mortality, we did not specifically evaluate minor adverse effects of the supplements such as nausea and vomiting among the mothers and newborns.

**Figure 1:**
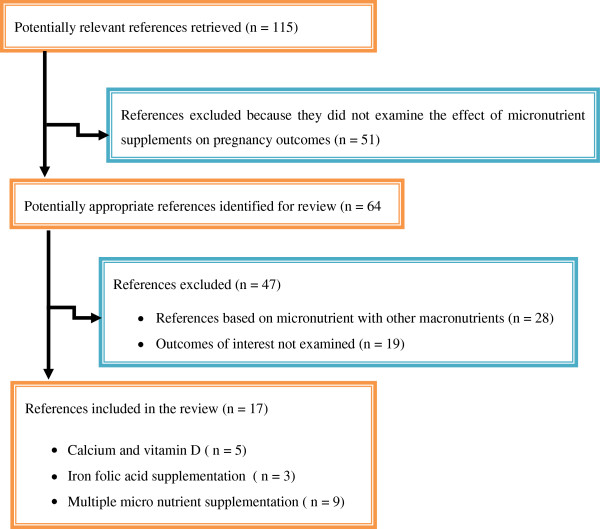
Selection of studies included in systematic review of randomized controlled trials and observational studies on maternal multiple micronutrient supplementation and pregnancy outcomes.

In the primary search 115 records were found. After exclusion of studies/reviews which did not examine the effect of micronutrient supplements on pregnancy outcomes duplicated and outdated (published before 2005) data, 64 articles were selected. During the second selection we evaluated 17 of them as potentially relevant articles considering the effect of multiple micro nutrient supplementations on pregnancy and birth outcomes. Studies that failed to meet our criteria were not taken into consideration (Figure 1).

We defined a “multiple micronutrient supplement” as a single tablet containing more than three different micronutrients; however micronutrient-fortified powders, foods and beverages were not included in the definition.

## Result and discussion

We identified 115 potentially relevant articles for detailed review (Figure 1). We excluded 51 articles during initial screening because they were not directly referring the effect of micronutrients on pregnancy. The remaining 64 articles were again analyzed for their internal consistency with the research objectives and some 47 of them were found not relevant and/or incomplete, hence excluded for the second time. The remaining 17 of were considered for final review and analysis. From these finally accommodated articles, five of them were related to the effect of Calcium and vitamin D supplementation, three of them were about Iron and iron folic acid supplementation, and the remaining majority [9] articles reviewed were about the effects of MMN supplementations on pregnancy and its outcomes.

Though some degrees of variation by type and amount of micronutrients supplied are observed, the results of the final analysis of the most articles reviewed, favored the supplementation of micronutrients to pregnant mothers.

The systematic review of clinical trials by Buppasiri P et al. from Thailand, with the objective of determining the effect of calcium supplementation on maternal, fetal and neonatal outcomes revealed that Calcium supplementation is associated with a significant protective benefit in the prevention of pre-eclampsia and improving mean infant birth weight. The same study also confirmed lack of additional benefits for calcium supplementation in prevention of preterm birth or low infant birth weight [12].

Another study with a similar finding by Hofmeyr GJ et al. concluded that Calcium supplementation appears to almost halve the risk of pre-eclampsia, and to reduce the rare occurrence of the composite outcome ‘death or serious morbidity’, with no other clear benefits, or harms [13].

The thesis based study by Melody B. on maternal vitamin D status during pregnancy as a predictor of offspring bone mass at three years of age shows that maternal vitamin D status during pregnancy did not predict for the child at 3 years of age [14]. Another systematic review by Bruce W. on dietary vitamin D requirements during pregnancy and lactation recommends further studies to determine optimal vitamin D intakes for pregnant and lactating women as a function of latitude and race [15].

Similarly, the study by Dror DK et al. shows the existence of recent evidence supporting the role of maternal vitamin D status, particularly early in pregnancy, in modulating the risk of pregnancy complications and in sustaining fetal growth, bone development, and immune maturation [16].

On the other hand, according to the findings of the many of the final articles reviewed; though few disfavored the beneficial outcomes of supplementing MMN to pregnant mothers; majority of the others in one way or another supported the presence of significant association and benefit of MMN supplementation with at least one birth outcome.

Among the studies against the added role of supplying MMN to pregnant mothers, the study by Parul Christian, which was about the Effects of alternative maternal micronutrient supplements on low birth weight in rural Nepal, revealed that Antenatal folic acid-iron supplements modestly reduce the risk of low birth weight but MMN confer no additional benefit over folic acid-iron in reducing this risk [17,18]. Another Indian study by Umesh Kapil et al. also said MMN Supplements will not Reduce Incidence of Low Birth weight and other pregnancy outcomes [19]. The Hungarian RCT study by Czeizel AE also revealed that Periconceptional multivitamin supplementation increased fertility but had no significant effect on the rate of different groups of fetal deaths, low birth weight and preterm birth in singletons [20].

Conversely, the remaining RCT and observational studies reviewed from various parts of the world confer that oral supplementation of MMN for pregnant woman benefits the mother and the growing fetus as well as the newborn.

In line with this, the reviews and RCT studies by Prakesh S., Zulfiqar A Bhutta, Michael B Zimmermann and other scholars across the globe unanimously reported that supplementing pregnant mothers with MMN immensely benefits both the mother and the growing fetus in reducing maternal morbidity conditions and morbid neonatal birth outcomes [21-23].

Similarly, the systematic review by Prakesh S. et al. also indicates that prenatal MMN supplementation is associated with a significantly reduced risk of low birth weight and with improved birth weight when compared with iron–folic acid supplementation. However, there was no significant effect of MMN supplementation on the risk of preterm birth or small-for-gestational-age infants [21,22].

Additionally, the review by Zulfiqar A Bhutta et al. also provides evidence on significant benefit of MMN supplementation during pregnancy on reducing SGA births as compared to iron-folate, with no significant increase in the risk of neonatal mortality in populations where skilled birth care is available and majority of births take place in facilities [22].

Furthermore, other review by Kosuke Kawai et al. discusses the safety, efficacy and effective delivery of maternal micronutrient supplementation requiring further research. The other study by Dr. Kathleen Abu-Saad on the other side, declares that maternal nutrition is a modifiable risk factor of public health importance that can be integrated into efforts to prevent adverse birth outcomes, particularly among economically developing/low-income populations [24,25].

On the other hand, RCT by Philip N Baker et al. revealed that Poor micronutrient intake and status increases the risk of SGA births in pregnant adolescents and hence mothers should get prenatal micronutrient supplementation in addition to the customary iron folic acid supplementation [26].

In addition, in a poor population, the effects of maternal MMN supplementation on the fetus persisted into childhood, with increases in both weight and body size. Maternal MMN supplementation might therefore be an important part of overall strengthening of prenatal-care programmes [26-28].

Therefore, it is clear to see that given comparability of impacts on maternal anemia, the decision to replace iron-folate with MMN during pregnancy may be taken in the context of available services in health systems and birth outcomes monitored.

## Conclusion

It was possible to learn that almost all available studies of RCT and other observational studies conducted in various parts of the world revealed that it is beneficial for the mother and the neonate and even to childhood with supplementation of MMN than ordinarily iron folic acid supplementation; nonetheless, there are still some equivocal findings related to the effect of MMN supplementation on the risk of preterm birth, small-for-gestational-age infants and neonatal mortality. Therefore, further studies at various combination and doses of micronutrient supplements particularly in areas where there is high prevalence of malnutrition are recommended.

## Competing interests

The authors declare that they have no competing interests.

## Authors’ contributions

TA developed the review parameters and secured support. He also undertook partial literature search, data extraction and analysis. HT made detailed and extensive literature search and involved in the write up and synthesis of the findings. He also reviewed and standardized the study. Both authors read and approved the final manuscript.
